# A Reference Architecture for Data-Driven and Adaptive Internet-Delivered Psychological Treatment Systems: Software Architecture Development and Validation Study

**DOI:** 10.2196/31029

**Published:** 2022-06-20

**Authors:** Suresh Kumar Mukhiya, Yngve Lamo, Fazle Rabbi

**Affiliations:** 1 Western Norway University of Applied Sciences Bergen Norway; 2 University of Bergen Bergen Norway

**Keywords:** software architecture, adaptive system, IDPT system, health care systems, ICBT, adaptive strategies, personalized therapies, reference architecture

## Abstract

**Background:**

Internet-delivered psychological treatment (IDPT) systems are software applications that offer psychological treatments via the internet. Such IDPT systems have become one of the most commonly practiced and widely researched forms of psychotherapy. Evidence shows that psychological treatments delivered by IDPT systems can be an effective way of treating mental health morbidities. However, current IDPT systems have high dropout rates and low user adherence. The primary reason is that the current IDPT systems are not flexible, adaptable, and personalized as they follow a fixed tunnel-based treatment architecture. A fixed tunnel-based architecture follows predefined, sequential treatment content for every patient, irrespective of their context, preferences, and needs. Moreover, current IDPT systems have poor interoperability, making it difficult to reuse and share treatment materials. There is a lack of development and documentation standards, conceptual frameworks, and established (clinical) guidelines for such IDPT systems. As a result, several ad hoc forms of IDPT models exist. Consequently, developers and researchers have tended to reinvent new versions of IDPT systems, making them more complex and less interoperable.

**Objective:**

This study aimed to design, develop, and evaluate a reference architecture (RA) for adaptive systems that can facilitate the design and development of adaptive, interoperable, and reusable IDPT systems.

**Methods:**

This study was conducted in collaboration with a large interdisciplinary project entitled INTROMAT (Introducing Mental Health through Adaptive Technology), which brings together information and communications technology researchers, information and communications technology industries, health researchers, patients, clinicians, and patients’ next of kin to reach its vision. First, we investigated previous studies and state-of-the-art works based on the project’s problem domain and goals. On the basis of the findings from these investigations, we identified 2 primary gaps in current IDPT systems: lack of adaptiveness and limited interoperability. Second, we used model-driven engineering and Domain-Driven Design techniques to design, develop, and validate the RA for building adaptive, interoperable, and reusable IDPT systems to address these gaps. Third, based on the proposed RA, we implemented a prototype as the open-source software. Finally, we evaluated the RA and open-source implementation using empirical (case study) and nonempirical approaches (software architecture analysis method, expert evaluation, and software quality attributes).

**Results:**

This paper outlines an RA that supports flexible user modeling and the adaptive delivery of treatments. To evaluate the proposed RA, we developed an open-source software based on the proposed RA. The open-source framework aims to improve development productivity, facilitate interoperability, increase reusability, and expedite communication with domain experts.

**Conclusions:**

Our results showed that the proposed RA is flexible and capable of adapting interventions based on patients’ needs, preferences, and context. Furthermore, developers and researchers can extend the proposed RA to various health care interventions.

## Introduction

### Background

Internet-delivered psychological treatment (IDPT) systems are software applications that offer psychological therapies or treatments through the internet. In our study, we focused on treatments based on evidence-based psychological therapy [1]. *IDPT systems* borrow core ideas from learning management systems and other content management systems (CMSs). However, IDPT systems are more inclined toward the health care domain and have a principal perspective of helping patients cope with their psychological problems. IDPT systems have 2 types of content: psychoeducational materials and treatment exercises.

### Problems With Current IDPT Systems

We attempt to address 2 problems associated with the current IDPT systems.

First, despite evidence that web-based interventions can be effective means for mental health morbidities, most of the current IDPT systems are tunnel based, inflexible (unable to adapt according to user needs, preferences, and context), and noninteroperable [2-4]. These restrictions cause a high dropout rate; less personalization; and hence, low user adherence [3,4].

Second, IDPT systems targeting different psychological issues (such as depression, anxiety, bipolar disorder, schizophrenia, and others) have many similarities in psychoeducational materials, intervention structures, and assessment techniques. However, because of the lack of standard documentation, established frameworks, and clinical guidelines, several forms of IDPT models exist. As a result, developers and researchers tend to reinvent their versions of IDPT systems, making them more complex and less interoperable [5]. Interoperability in such IDPT systems is essential for exchanging information from one system to another. With the prevalence of ambient intelligence, several Internet of Things devices have been connected to assess, monitor, and guide patients. These devices require communication with each other. In addition, people migrate from one geographic area to another. Consequently, there is a need to share data from one software system to another.

### Objective

To address the issues associated with the current IDPT system, we conducted this study with 2 objectives.

The first objective was to create a reference architecture (RA) [6] of an *adaptive IDPT system*, which can personalize the treatments according to patients’ needs and support adaptability, interoperability, reusability, scalability, security, and modifiability. *Adaptability* is the ability of a system to accommodate treatments based on patient needs, preferences, and context. *Interoperability* is the ability of a system to exchange information correctly and use the information being exchanged. We use reusability in two contexts: (1) the ability to use the treatment for other types of mental health care and (2) the ability to use the component of the IDPT system. *Scalability* is the ability of a system to grow with the number of patients, data, or other factors increases. *Security* is the ability of a system to communicate safely when considering malicious attacks. *Modifiability* is the ability to evolve and maintain systems.

On the basis of the proposed RA, the second objective was to create an *open-source framework* [7] that can be used to develop an adaptive IDPT system. The *open-source framework* was created to aim to (1) improve development productivity; (2) facilitate communication with domain experts; and (3) improve the quality of user interfaces (UIs), user interactions, and user experiences.

### The Need for Adaptive IDPTs

IDPTs have surfaced and become one of the most commonly practiced and widely researched forms of psychotherapy [8]. The evolution of IDPTs, coupled with the exponential growth of internet access worldwide, has the potential to reshape the landscape of mental health care. Despite the evolution of IDPT, several patients with mental health issues remain untreated [9,10]. Obstacles to receiving treatment for mental health problems include long waiting lists, limited access to therapy and psychiatric medications, perceived stigma of seeking help, and treatment costs [3,10,11]. IDPT systems have been proposed as a solution to bridge this treatment gap. IDPT removes several barriers to traditional face-to-face therapy, which hinders most patients from receiving efficient psychiatric care [12]. The use of IDPT tools can enhance mental health in several manners:

1. IDPT is available and accessible from anywhere through an internet connection [13].
2. The temporal aspects of accessing the treatments can be substantially improved.
3. The scalability of IDPT can drastically enhance the functional capacity of the care [14]; for example, multiple patients can receive treatment at the same time.
4. IDPT makes the treatment cost-effective for individuals who do not have insurance or cannot afford out-of-pocket fees for treatment.
5. IDPT removes the discomfort and stigma-related issues associated with face-to-face approaches [14].

**Figure 1:**
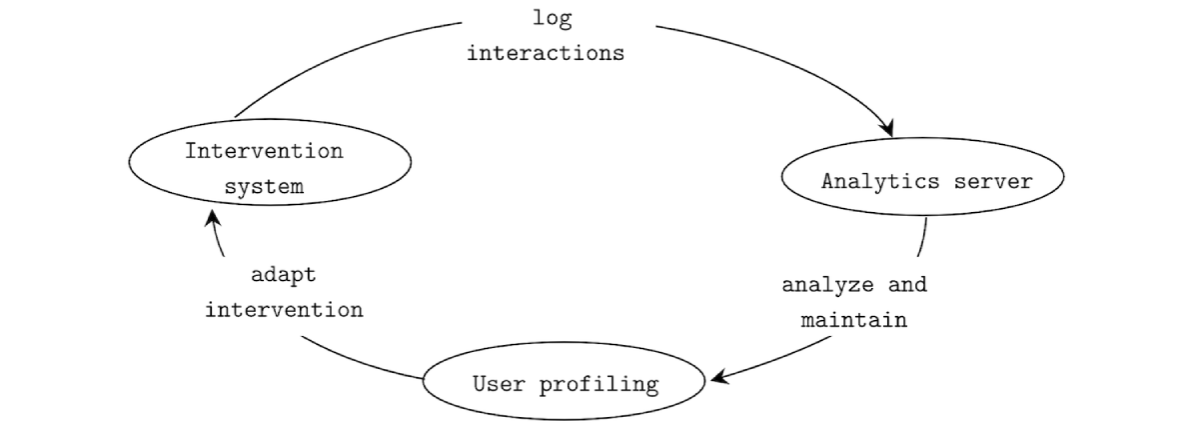
The figure depicts our proposed model of a data-driven adaptive internet-delivered psychological treatment system. The patients interact with the intervention, and an analytics server captures those interactions. On the basis of the logged data analysis, a process referred to as user profiling maintains an up-to-date user model to provide the adaptive effect.

**Figure 2:**
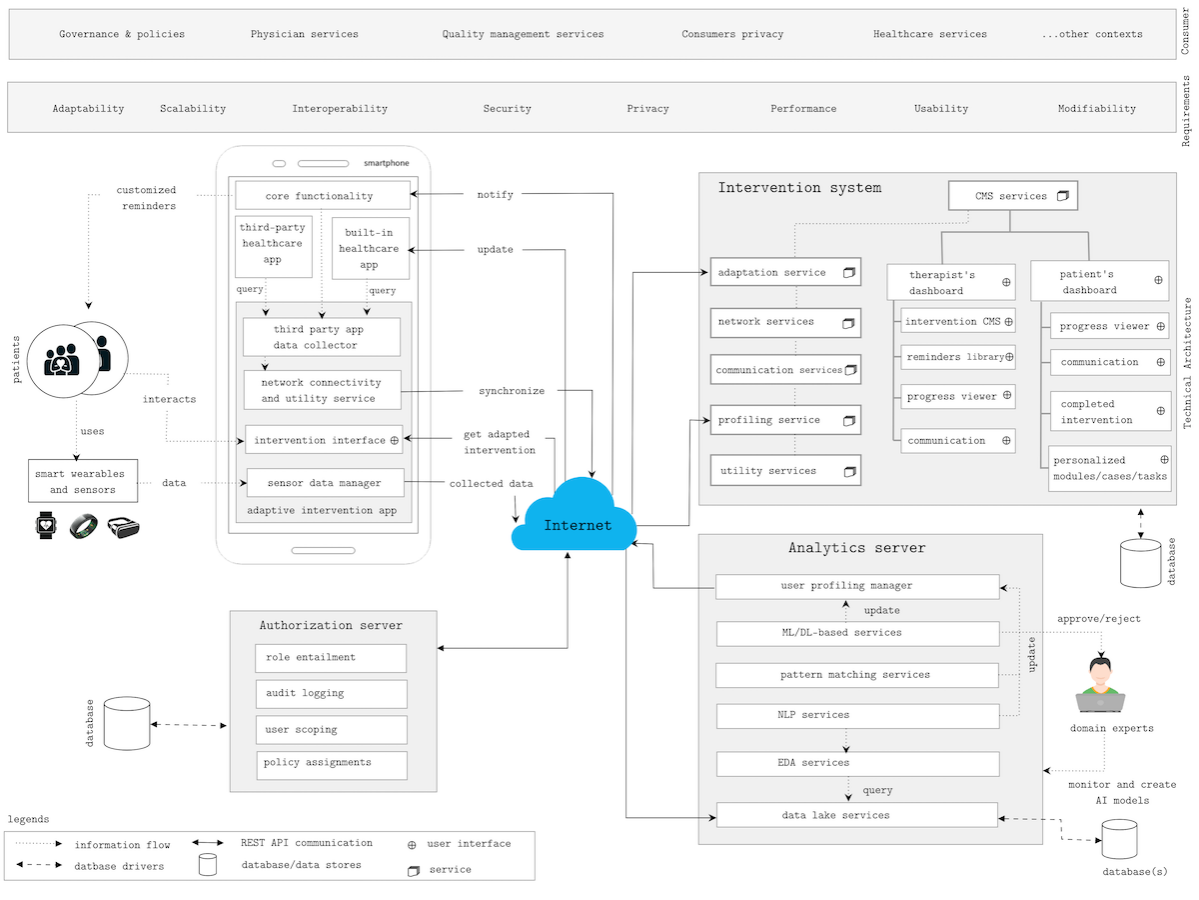
Reference architecture of data-driven adaptive internet-delivered psychological treatment system. AI: artificial intelligence; API: application programming interface; CMS: Content Management System; DL: deep learning; EDA: exploratory data analysis; ML: machine learning; NLP: natural language processing; REST API: RESTful API.

**Figure 3:**
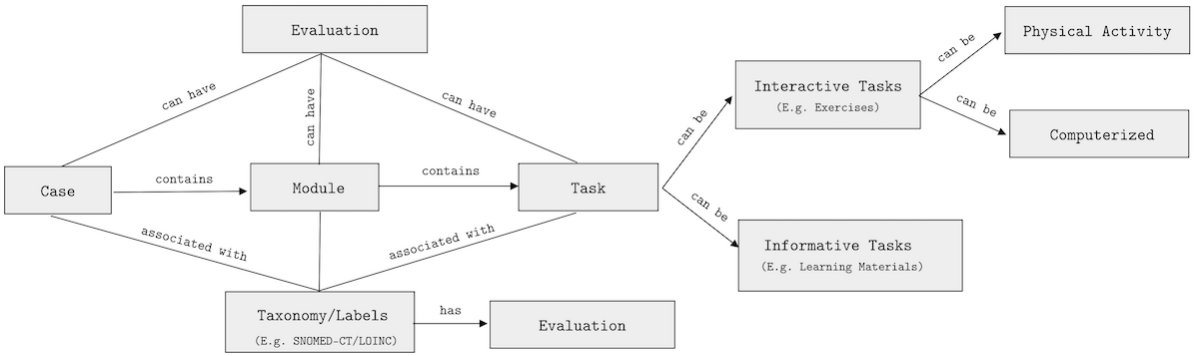
The figure depicts the conceptual model of any intervention that is always associated with a case. A case contains ≥1 module. Each module has ≥1 task, which can be learning materials or exercises. An exercise can be physical or computerized activities. LOINC: Logical Observation Identifiers Names and Codes; SNOMED-CT: Systematized Nomenclature of Medicine–Clinical Terms.

Despite this evidence, most current IDPT systems are not adaptive and have poor interoperability. These restrictions cause a high dropout rate; less personalization; and hence, low user adherence. Hence, there is a crucial need for an intervention system that can help personalize treatments and increase user adherence. Current learning management systems and CMSs are not designed to capture information on mental symptoms, and they do not monitor treatment progress and relevant data. Moreover, they cannot address the need for personalization and interoperability. To address these intrinsic requirements, we propose a new RA and evaluate it by developing an open-source framework based on the RA. The proposed architecture relies on the user profiling technique for personalization (Figures 1 and 2) and ontological labeling for interoperability (Figure 3).

### How We Propose to Increase Adaptivity in IDPT Systems

Figure 1 shows the interaction model of the proposed data-driven adaptive IDPT system. Patients interact with the *intervention system* (see the *Intervention System* section), and an *analytics server* (see the *Analytics Server* section) stores these interactions. In our RA, an analytics server refers to third parties or self-contained services that contain data stores to collect a large amount of data and provide data analytics services such as pattern matching, natural language processing (NLP) service, exploratory data analysis service, machine learning (ML) and deep learning (DL) services among others. On the basis of the analysis of logged data, a process referred to here as *user profiling* (see the *User Profiling* section) maintains an up-to-date user model. A user model is used to provide an adaptive effect. Adaptive systems behave differently for different users. The decision on how the system should behave for any particular user is based on a user model. A user model is a detailed representation of an individual user’s information that is associated with an adaptive system. User preferences and needs are dynamic. Hence, it is essential to create, maintain, and update the user model. An adaptive system accumulates data using two distinct approaches to create and maintain an up-to-date user model: (1) implicitly by observing user interactions and (2) explicitly by requesting direct input from the user. This process is referred to as *user profiling*. The essence of the adaptation effect that a system can deliver depends on the nature of the user model’s information. Hence, in this study, we aimed to present a framework based on user profiling to provide different adaptation effects.

### Contributions

The contributions of this study are 2-fold.

First, we propose an RA for an adaptive IDPT system that provides different adaptation levels based on user profiling. To the best of our knowledge, this novel study is a pioneer in creating, evaluating, and publishing an RA in this domain.

Second, to evaluate the proposed architecture, we created an open-source framework that can be easily extended to several health care interventions. We envision promoting open-source development by creating a proof-of-concept prototype based on the proposed architecture.

### Related Work

Grua et al [15] presented an RA for personalized self-adaptive eHealth apps. The proposed RA was envisioned to personalize and self-adapt interventions and increase user engagement with artificial intelligence (AI) applications. The proposed RA uses the Monitor-Analyze-Plan-Execute loop and is primarily targeted at mobile apps. Moreover, the RA follows a client-server architecture and assumes a self-containing, fully flexible AI-enabled back-end system. Such a self-containing back end is neither scalable nor flexible, especially for small-scale health care providers. Health care providers specialize in their domain and dedicate services such as AI, CMS, authentication, and authorization to third parties. For example, in Norway, the health care system relies on a level 4 security system such as BankID [16] for authentication and authorization and AI services from Microsoft Azure or Amazon Web Services. Not all services are coupled into a self-containing system. In such a scenario, *Service-Oriented Architecture* (SOA) is suitable, similar to what we provided in our RA. Unlike their RA, our RA focused on a loosely coupled intervention system that incorporates intervention authorizing services, user profiling services, adaptation services, and others.

WSO2 [17] presents a layered structure that targets scalability and security. On the one hand, the architecture is abstract and domain independent and lacks a specific mechanism to adapt the intervention according to the patient’s needs. On the other hand, Wartena et al [18] outlined the RA of a personal telehealth ecosystem referred to as Continua. The proposed RA uses the end‐to‐end architecture as a design guideline to support interoperability. Continua identifies personal area network devices for communication around a person, local area network devices for communication around a location, wide area network devices for communication around a home and office, and health reporting network devices for communication around enterprise systems such as hospitals, telehealth services, and others. In addition, this study reported how these devices could communicate using associated protocols, promoting interoperability. However, the architecture was abstract, did not address other software quality attributes besides interoperability and security, and did not report how one adapts interventions or personalizes health services.

Rodriguez [19] presented a detailed RA regarding Health care Supportive Homes, a particular type of Ambient Assisted Living (AAL) domain. The proposed RA provides detailed guidelines that can be used to achieve software quality attributes such as interoperability, reusability, security, safety, performance, and reliability. The study reported a detailed and stepwise recommendation for creating a reusable RA. Similar to Continua, Hanke et al [20] presented a *universAAL* reference model for establishing a cross-application platform for AAL. However, both the RAs were specific to the AAL domain. Moreover, they fit the psychological perspective to adapt interventions according to the patient’s needs. Mukhiya et al [4] conducted a systematic literature review to identify adaptive elements (content, presentation, feedback message, assessment, activities, reminders, exercises, and reports) of an IDPT system for mental health disorders. The study concluded that most current IDPT systems attempt to adapt feedback messages to patients from therapists. The study reported the lack of an open-source framework for creating adaptive IDPT systems.

Researchers have attempted to theorize user profiling for adaptive web, personalization, and intelligence systems [21-24]. Similar to the studies by Brusilovsky and Millan [21] and Schiaffino and Amandi [24], we considered interest, knowledge, background, goals, individual tasks, and context as essential user profile components. However, in addition to these, we considered several other attributes, including temporal profile, lingual profile, user level, and intervention profile. Furthermore, we modeled these attributes in the proposed framework and illustrated how they could facilitate psychological intervention personalization.

## Methods

As a part of the INTROMAT (Introducing Mental Health Through Adaptive Technology) project (see the *Acknowledgment* section), we envisioned developing an adaptive system to offer personalized and customized treatments for patients with mental and neurological disorders. To satiate this goal, we started with the procedures described in the following sections.

### Evaluating State-of-the-Art Digital Psychological Treatments Systems Concerning the Current Treatment Requirements

We included usability and universal design principles to evaluate current IDPT systems and publish our findings in this study [25]. Our findings indicated that despite satisfactory treatment results and proven clinical effects, in general, the systems have several issues regarding usability, universal design, and outdated technology.

### Collecting Recommendations From Research by Conducting a Systematic Literature Review

We conducted a systematic literature [4] review to (1) inspect and identify the main adaptive elements of an IDPT system, (2) find its information architecture, and (3) determine how adaptation influences the efficacy of IDPT on mental health treatments. The review suggested that adaptive IDPT has the potential to enhance intervention outcomes and increase user adherence. However, current IDPT systems are tunnel based and do not offer personalized treatment according to user needs. To comprehend how usability is addressed and measured in mobile health interventions for mental health problems, we conducted a systematic literature review [26]. We publish our findings from the perspective of computer science and human-computer interaction in this study [26]. Most studies described their methods as trials, gathered data from a small sample size, and conducted a summative evaluation using a single questionnaire, which indicates that usability evaluation was not the main focus.

### Collaborating With Domain Experts and Stakeholders to Comprehend Actual User Needs

Technical domain experts included academicians and industry workers from software engineering, human-computer interaction, AI, and health informatics. Health care domain experts were personal consultants for several mental and neurological disorders. We followed the Domain-Driven Design (DDD) architectural style [27] to model and create the adaptive IDPT framework as these systems involve creating software programs that facilitate the delivery of psychological health care treatments over the internet. Psychological treatments fall under the complex domain, and the development of software systems requires thoughtful collaboration between domain and technical experts. When the domain is complex, it is difficult for designers and developers to build the software. In such cases, developers must steep themselves into the domain to build up their business knowledge. However, most developers do not have much interest in learning about a specific domain in which they are working. In such use cases, the DDD method comes to the rescue.

With the help of domain experts and A technical team, we created the proposed RA. To evaluate the RA, we developed an open-source framework that is presented in this study. We have open sourced the initial prototype under a Massachusetts Institute of Technology License, where everyone is permissible to extend the framework without any consequences. The framework follows the SOA for communication. The server side of the framework follows the Back end for Front end architecture pattern. We followed the Test-Driven Development [28] during framework development. To evaluate the RA’s and proposed open-source framework efficacy, we continuously extended the framework for several health care issues, performed randomized controlled trials (RCTs), conducted usability evaluations, and enhanced the system.

### Ethical Considerations

This study is a part of the INTROMAT (Introducing Mental Health through Adaptive Technology). As a part of this project, the study was exempted from obtaining ethical approval and the authors have been permitted to publish their findings and research without external approval.

## Results

### Overview

We use the term *RA* concerning the context and definitions provided by Cloutier et al [6]. As suggested in this study, our proposed RA encompasses three essential questions: (1) what (ie, the intervention system and its components), (2) why (ie, to adapt and personalize the intervention to enhance user adherence and reduce dropouts), and (3) how (ie, by creating detailed user profiling and using AI and other adaptive strategies to adapt the intervention). One of the reasons for this study is to disseminate the proposed RA, the open-source framework, and ideas for constructing adaptive web applications for health care treatments. To meet the objective of this study, we contextualized a web application at a high level without focusing on specific expertise. From this contextualization, we designed models to comprehend application behavior. Figure 2 shows the RA of the adaptive IDPT system. As the vision is a high level of abstraction, we eliminated constraints related to design, external stakeholders, and others from the model. The RA constitutes 4 major components*: authorization server*, *mobile client*, *intervention system*, and *analytics server*.

### Authorization Server

The authorization server [29] is an OpenID Connect–compliant web server [30] with the ability to authenticate patients and grant authorization access tokens. Moreover, the authorization server manages the scopes and permissions of the patients, introspects tokens, entails roles and permissions, audits logs, assigns policies, and requests the intervention system. Our open-source framework included a stand-alone authentication server. However, as the adaptive system follows the SOA architecture, any third-party authentication server can be easily integrated with the framework. For example, in Norway, the use of BankID [16] for authentication or authorization is common.

### Mobile Client

The mobile client is the host where the adaptive intervention app (mobile health app) is installed. The mobile client app contains a *third-party app data collector, network connectivity and utility service, intervention interface,* and *sensor data manager*. The *third-party app data collector* is responsible for communication with third-party apps, health care apps, and built-in health care apps to collect health care data. The *sensor data manager* collects sensors data from Internet of Things devices. The *network connectivity and utility service* are responsible for sending these health care and sensor data to *data lake services* in the analytics server. The mobile client incorporates *intervention interfaces* that allow patients to interact with adapted interventions and communicate with the therapists.

### Analytics Server

Conceptually, the analytics server has two parts: (1) the structural part of building a user profile and (2) the analytics method of feeding information to the profile. The analysis servers (Figure 2) incorporate analytical software as a service application programming interface (API). These services take the data as input, detect patterns, and provide a detailed analysis. Although there were several possibilities for the types of algorithms used for data analytics, in the adaptive IDPT context, we aimed for the following core functionalities:

1. *Data lake services* accumulate both sensors and intervention data.
2. *Exploratory data analysis services* help with data cleaning, preparation, exploration, and visualization.
3. *NLP services* help in building, evaluating, and detecting patterns in the textual data set. For example, when patients interact with an intervention, they write some texts as part of computerized exercises. These texts exhibit keywords that express the patient’s current state or emotions. It is possible to send these texts directly to available NLP APIs such as Google NLP, obtain the sentiment and tone of the texts, and detect the presence of depression-related keywords [31]. In this study [31], we demonstrate how we can exploit the NLP technique to extract depression symptoms from patient-authored texts.
4. *Pattern matching services* can reveal several associations, correlations, and hidden patterns in the sensor and intervention data. For example, Sharma et al [32] presented a large-scale analysis of the engagement patterns of 35 million posts on 2 popular web-based mental health platforms: TalkLife and Reddit. This study demonstrates that the proposed framework of the engagement patterns enables informative evaluations and analysis of web-based support platforms.
5. *ML and DL-based services* constitute ML *and* DL algorithms. Data from sensors and interventions can be used to predict early dropout rates and personalize interventions. Once a model has been developed, trained, and evaluated, domain experts can evaluate it for approval. For example, Bremer et al [33] outlined the use of ML techniques to predict dropout in insomnia interventions. Similarly, Nemesure et al [34] proposed an ML approach to predict the presence of generalized anxiety disorder and major depressive disorder.
6. The *user profiling manager* is envisioned to use the analyses and predictions made by NLP services, pattern matching services, and ML and DL services to build a comprehensive profile for the patient.

### Intervention System

#### Overview

The intervention system (Figure 2) is ≥1 web application communicating via *web services* such as RESTFul API or GraphQL API. This comprises several services. *CMS services* facilitate a therapist’s dashboard (intervention creator, reminder library, progress viewer, communication channels) UI and a patient’s dashboard UI (progress viewer, communication channel, history, next or upcoming intervention modules, or tasks). *Adaptation services* provide a rule-based engine for building adaptation rules based on user profiles. *User profiling services* maintain the user profiles. Similarly, *communication services* create a communication channel between patients and therapists. *Network* and *utility services* handle internet connectivity logic and other utility-oriented tasks. One may separate these components and communicate using a microservice architecture [35]; however, the intervention system components are monolithic for this open-source framework. Figure 3 depicts a conceptual model of the intervention. An intervention is a psychological treatment or therapy delivered through IDPT systems. An IDPT system refers to software that facilitates the creation and delivery of and interaction with psychological therapy through the internet. These include web applications, mobile apps, augmented reality, and virtual reality applications.

#### Components

Interventions generally comprise cases, modules, tasks, and taxonomies (labels). In this section, we explain these components, their underlying assumptions (Textbox 1), and their constraints.

**Different assumptions that were considered when designing the open-source framework.**

**Components and assumptions**

**Case**

- A case contains at least one module.
- A case can have ≥1 evaluation criterion.
- A case can have user inclusion and exclusion criteria.

**Modules**

- A module contains at least one task.
- A module can belong to ≥1 case.
- A module can depend on other modules.
- A module can have ≥1 evaluation criterion.

**Tasks**

- A task can have subtasks.
- Each task can have ≥1 evaluation criterion.
- The evaluation criteria of a task are the overall evaluation of the subtasks.
- Tasks can have dependency but cannot have their own dependency.

#### Cases

**Figure 4:**
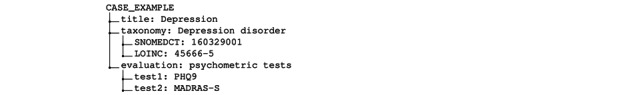
Example data structure of a case.

Typically, IDPTs target ≥1 case such as depression, social anxiety, bipolar disorder, attention-deficit/hyperactivity disorder, or other health issues. An example of a *case* is shown in Figure 4.

#### Modules

Each case contains ≥1 module that focuses on any particular dimension of the case. For example, in the case of depression, there can be modules for understanding and monitoring emotions, behavioral activation, identifying automatic thoughts, and others. A specific module can be part of ≥1 case. The modules can have dependencies that specify their ordering.

#### Tasks

In turn, each module can include ≥1 task. A task can be learning materials (informative task) or an exercise (interactive task). *Informative tasks* provide learning materials on mental health issues (cases), symptoms, use cases, and several ways of managing them. The main objective of such informative tasks is to provide psychoeducation so that patients and their families can learn about symptoms, causes, and treatment concepts; patients can comprehend the self-help program and steps required to manage their illness; and patients can correlate their situations with others who have similar issues, which helps to ventilate their frustrations.

**Figure 5:**
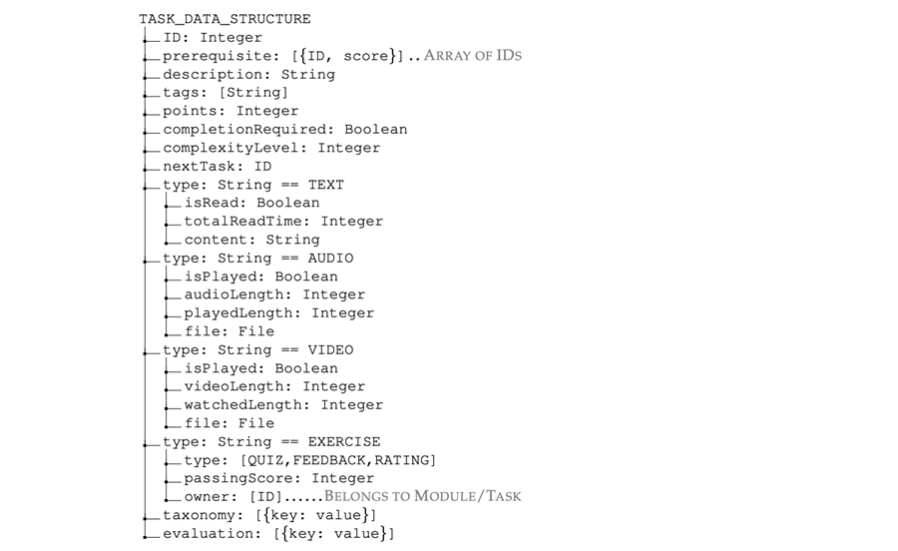
Data structure of a task.

These informative tasks are in the form of reading (text), listening (audio), graphics, presentations, and watching (video). In contrast to informative tasks, *interactive tasks* involve user interaction, often in the form of exercises and psychometric tests. These exercises can be *physical activities* or *computerized tasks*. Examples of physical activities include physical workouts and mindfulness exercises such as breathing exercises, walking certain distances, stretching, or physically performing other activities. Examples of computerized exercises involve filling in blanks, answering (questions and answers), multiple-choice questions, and feedback. The feedback forms comprise the use of free text, rating systems, or multiple-choice questions. The minimal data structure of a task and its types are illustrated in Figure 5.

#### Taxonomy or Labels

Each case, module, and task is associated with a label or taxonomy. As cases, modules, and tasks form the hierarchical structure, these taxonomies provide ontological structures for adaptation.

#### Constraints

A task or module may have ≥1 constraint. These constraints determine the states (see the *States of Intervention Components* section) of the task and module. As illustrated in Figure 5, a task or module can have the following constraints:

1. Prerequisite, which is a list of tasks required to be completed before the task is active
2. Next task, which is a task that can have a restrictive follow by selecting the next available task
3. Completion required, which is a Boolean value that represents that a user must complete the task if truthy
4. A passing score on an exercise of a quiz type determines whether an exercise is complete

#### States of Intervention Components

**Figure 6:**
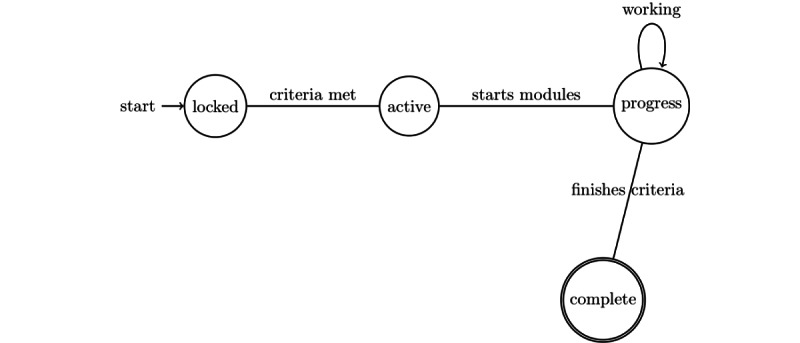
States of cases, modules, and tasks of an internet-delivered psychological treatment system.

All the cases, modules, and tasks can have four different states, as shown in Figure 6:

1. *Locked:* An entity is locked if its evaluation criteria are not fulfilled or if a dependent entity is not completed.
2. *Active:* An entity is active as soon as the evaluation criteria are matched or its dependent entity is completed.
3. *Progress:* An entity is in progress if it is active, but all the evaluation criteria have not been completed.
4. *Complete:* An active entity is marked as complete if all evaluation criteria are completed.

### User Profiling

#### Overview

A profile is a description of an actor containing the necessary facts about the individual. In an adaptive IDPT context, a user profile (or user model) holds essential facts about an individual patient. The process of inferring unobserved data about users from observable data about them (ie, their actions or interactions) is referred to as user profiling [36]. The primary motivation for building user profiling is that users differ in their preferences, interests, backgrounds, goals, cognitive skills, and other attributes. Discovering these differences is essential for presenting users with personalized or adapted services. In an adaptive IDPT system context, user profiling aims to provide an adaptation effect; that is, to behave differently for different users [21]. As mentioned previously, we envision applying user profiling as a fundamental basis for adaptation. Hence, we discuss user profiling in this section and discuss how we can use such profiling to adapt interventions in the *Scenario-Based Evaluation for Adaptation Support* section. It is essential to note that user profiling can be based on a distributed architecture. Hence, the data-driven adaptive system presented in Figure 1 follows the SOA. An adaptive system tends to find the most relevant information to the user’s interests and presents the information in the right form so that the user may perceive its relevance. A user profile typically powers the discovery of such relevancy and its ranking. A user profile can contain several components (see the *Components of a User Profile* section), such as user interest, knowledge, background, goal, individual traits, and user context. An adaptive system can create and maintain the user profile explicitly and implicitly. We discuss these data acquisition methods in the *Methods of Collecting Information for the User Profile* section. Table 1 summarizes the different aspects of a user profile, including the components of a user profile, the form of representation in software, and the types of data stored in each of the component processes of obtaining data.

**Table 1:** Different components of use profiling techniques.

Attributes	Common representations	Types of data	Profiling approach
Interest	Weighted vector of keywords; topic hierarchies	News topics, webpage topics, document topics, work-related topics, and hobbies	Implicit or explicit
Knowledge	Scalar modeling; overlay modeling	Application domain	Implicit
Background	Stereotype modeling	Profession, job responsibilities, experience of work, and specific view on the domain	Explicit
Goals or tasks	Goal catalog approach	Goal of the work, information need, and learning goal	Explicit
Individual traits	Mixed approaches	Cognitive styles, personality traits, learning styles, and demographic	Implicit or explicit
Context	Set of name-value pairs	Platform, location, physical environment, social context, and affective state	Implicit

#### Components of a User Profile

##### Overview

The content of the user profiles varies according to the system’s domain and the software architect who designed the system. There are no specific standards that specify which components should be in a profile. Similar to the studies by Brusilovsky and Millan [21] and Schiaffino and Amandi [24], we categorized the user profile content into the components described in the following sections for our framework.

##### Interests

User interests affect their adherence to software systems. Hence, capturing user interests and attempting to personalize content based on their interests can be an effective means of boosting user adherence. A software system can represent user interest in two ways:

1. The weighted vector of keywords: For example, Lieberman et al [37] used term frequency and inverse document frequency to model user interests. In the term frequency and inverse document frequency technique, each word’s weight is computed by comparing the word frequency in a document against the word frequency in all documents in the corpus.
2. Topic hierarchies: A graph can express topic hierarchies where a node is a set of topic words representing a user’s specific interest. These types of representations are essential when modeling user interests and associated subtopics.

##### Knowledge

The user’s knowledge represents their understanding of the subject or domain. The user’s knowledge is a dynamic feature that increases or decreases over time. Therefore, a well-adaptive system should recognize a user’s current state of knowledge and tailor the user model accordingly. A software system can represent a user’s knowledge in two ways (Table 1):

1. Scalar modeling: Scalar modeling systems use quantitative scales (for example, 0 to 10) or qualitative scales (eg, excellent, very good, good, bad, poor, and none). However, formulating scalar values for user knowledge is challenging. Hence, scalar modeling has low precision.
2. Overlay modeling: In overlay modeling, the domain contains ≥1 subfragment. For each fragment, an overlay model stores the estimation of the user knowledge. The estimation can be binary (knows or does not know), qualitative (excellent, very good, good, bad, poor, or none), or quantitative.

##### Background

The user’s background constitutes information about their profession, job responsibilities, work experience, and a specific view of the domain. The most common representation format for a user’s background is stereotype modeling, as detailed background information is not essential. In stereotype modeling, a domain expert distinguishes the most common categories of users according to their background information and adapts the content presented to the user category. The system can also differentiate users by profession (student, medical person, teachers, and others), which implies both knowledge and responsibilities. Several adaptive systems use background information to adapt the content based on the background information of the user.

##### Goals

The user’s goal represents the purpose that the user desires to achieve from the system. The purpose can be information needs, learning goals, or the working of the applications (Table 1). These user goals are dynamic and change over time. Hence, it is essential to tailor the intervention according to the current user’s goal. The most common way of representing a user’s goal is to use the goal catalog approach, in which the system presents a predefined set of possible user goals. An adaptive system can recommend certain pages to the user based on a predefined set of goals [21] or adapt the content selected page [38].

##### Individual Traits

The user’s traits include cognitive styles, personality traits, learning styles, or demographic data. Several researchers have agreed on the importance of individual traits and their use in adaptation. Individual traits are stable features of a user, do not change at all or change over a long time, and can be extracted through specially designed psychological tests. Although *cognitive styles, personality traits*, and *demographic data* have been discussed in the literature, *learning styles* have been argued [39]. Various methods have been used to extract a user’s personality traits and cognitive styles and use them for adaptation.

##### Context

The prevalence of ubiquitous computing has attracted several researchers of the user’s context, such as location, social context, physical environment, and affective state, to tailor software systems. Most of the work on user context has focused on user platforms. For example, most of the studies attempted to adapt to make the system responsive [40] or tailor the content based on hardware, available software, and bandwidth. Affective contexts include physiological and mental contexts. The social context comprises the current user’s social aspects, such as information about friends, neutrals, enemies, neighbors, coworkers, and relatives. The most common way of storing the user context is in the form of a *key-value* pair.

#### Methods of Collecting Information for the User Profile

As mentioned previously, there are 2 ways of extracting the information required to build a user profile: *explicitly* or *implicitly*.

##### Explicit Information Extraction

A software system can extract profile components such as backgrounds, goals, and interests explicitly; that is, by asking users through UIs such as forms or feedback. Generally, users are not willing to fill in long forms to provide information about them; hence, they are optional. The information accumulated in this way includes demographic data such as age, job, and hobbies.

##### Implicit Information Extraction

Explicit method of user information has several challenges, including (1) users do not like to fill up long forms, (2) users do not always tell the truth when made obligated to feel forms, and (3) users who wish to fill up the form willingly may not know how to express their interests in words. Observing user interactions (time spent on the content page, bookmarked pages, amount of scroll, content viewed, video watched, and others) with a software system and logging these actions, we can obtain information about users through ML or data-mining techniques. A vital advantage of the implicit method is that we can log and analyze users’ changing interests, preferences, habits, and goals over time. These logs can help adapt the content or presentation according to the correct context of the user.

## Discussion

### Principal Findings

Architectural evaluation ensures that the architectural design decisions produced are the correct ones [41]. One of the RA evaluations aimed to analyze and verify that it addressed the problems identified in the current IDPT systems. We chose both empirical (case study) and nonempirical (expert evaluation and scenario-based method) evaluation techniques to analyze and verify the proposed RA and open-source framework. Two relevant options for scenario-based evaluation are the software architecture analysis method (SAAM) [42] and the architecture trade-off analysis method [43]. We chose to apply the SAAM method as we proposed RA and qualitatively evaluated it. Moreover, SAAM is suitable for assessing whether a given RA satisfies a specific system’s desired properties, whereas the architecture trade-off analysis method is more suited to determine the trade-off between architectural alternatives [43], as performed in the *Related Work* section.

### Scenario-Based Evaluation for Adaptation Support

#### Overview

The first SAAM method was to develop scenarios. This section presents how the proposed RA can perform different types of adaptations based on user profiling. These scenarios, along with the open-source framework’s initial prototype, served as scenarios for our evaluation process. The next step in SAAM is to describe the candidate RA outlined in the *Discussion* section. After that, it is necessary to identify the system quality attributes with the help of the developed scenario. The Software Quality Metrics section describes the identified software quality attributes extracted from our scenarios.

#### Content Adaptation

Content adaptation may involve two subcategories: (1) content materials adaptation, which involves deciding what content is the most relevant to the current user, and (2) content presentation adaptation, which involves determining how to present the selected content effectively to the current user.

#### Content Materials Adaptation

**Figure 7:**
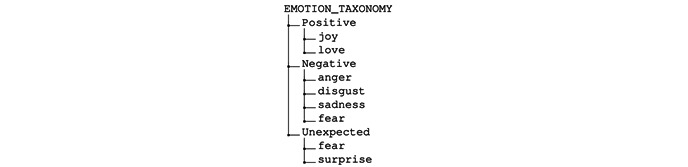
An example scenario of human emotion taxonomy.

The main task was to identify the most relevant content for a given user in their context and how to organize that content. User profile components such as *interests, preferences, background, knowledge*, and *goals* can help to select the most relevant content for a given user. The literature mentions 2 different approaches for adapting content: *page variants* and *fragment variants* [44]. Our RA supported both approaches for content adaptation. Moreover, with user profiling in place, we can perform *adaptation based on the metadata*. The details of the approaches are as follows:

1. In the *page variants approach*, different versions of each page are created using the CMS service (Figure 2). The chosen adaptive strategy [3] selects and presents the most suitable content to the user based on its current context and profile.
2. A page is divided into ≥1 fragment in the *fragment variants approach,* where each fragment corresponds to a self-contained element such as text, audio, video, paragraph, picture, or presentations. In an IDPT system, these fragments are authored by domain experts. These fragments are selected and presented to the user based on an appropriate adaptive strategy. Currently, most IDPT systems use rule-based adaptation mechanisms to predefine these fragments. However, once these fragments are predefined and labeled correctly, many pages can be automatically generated to cover a correspondingly large number of interaction contexts.
3. As illustrated in Figure 5, in metadata or taxonomy-based adaptation, a task can have several tags. These tags act as a list of controlled vocabulary sets that define several dimensions of a text. For example, a text can provide psychoeducation about different human emotions (*sad, happy, angry, disgust, sadness, joy, love,* and *surprise*). These controlled vocabularies can be abstracted to form a taxonomy and ontology related to any particular illness (Figure 7). For example, *joy* and *love* indicate positive emotions. Similarly, *anger*, *disgust*, *sadness*, and *fear* indicate negative emotions. While a user reads a text about *disgust*, these taxonomies can help recommend other tasks (audio, video, images, or activities) that exhibit similar labels as *anger, sadness*, or *fear* as they indicate negative emotions at a higher level. Moreover, *emotions* are associated with other mental health issues. Learning materials related to emotions for *depression cases* can also be used in *social anxiety cases*.

#### Content Presentation Adaptation

Let us assume that a task can be represented by the following modalities: audio, video, slides, and text. Here, we assume that each format preserves the semantic meaning of the original format. An IDPT system can personalize a content format based on user interests (see the *Interests* section) and goals (see the *Goals* section). An IDPT system can obtain user interests based on (1) a process mining technique, (2) explicitly asking users, (3) user interaction data, and (4) other data-mining techniques. Process mining can reveal the format of the content that a particular user interacts with the most. If a user spends more time watching videos, the system can present the next video format task. In addition to process mining, user interaction data can reveal the preferred content format for any particular user. These preferences and interests are stored in the user profile and are used for content format adaptation.

#### Reminders or Alert or Other Notifications Adaptation

**Figure 8:**
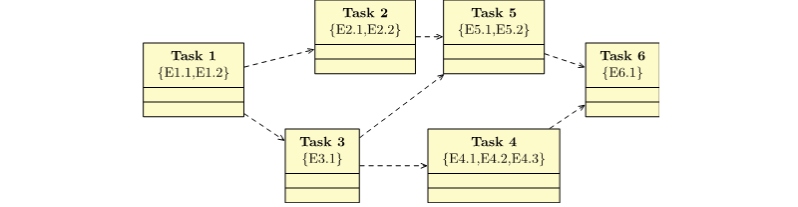
An example of different tasks inside a module in an internet-delivered psychological treatment system. Here, the dotted arrow denotes dependencies. For example, Task 2 is dependent on Task 1. Such dependency indicates that a patient cannot start Task 2 before Task 1. Each task has ≥1 evaluation criterion denoted as E.x.

Figure 8 illustrates an example of different tasks inside a module in a typical IDPT. As shown in the figure, *Task 2* and *Task 3* have *Task 1* as a dependency. This dependency means that a patient must finish *Task 1* before *Task 2* and *Task 3* are active for them. In addition, to complete *Task 1*, the patient must fulfill both evaluation criteria *E1.1* and *E1.2*. Once *Task 1* is completed, it is marked *complete,* and *Task 2* and *Task 3* are *active*. In addition, the IDPT system schedules an automatic alert or notification for the patient with a personalized message indicating the completion of the task and availability of the next tasks. As shown in Table 2, the process of customized alerts or notifications can be adapted based on the task status. The alert or notification adaptation scenario presented here is an example and can be extended based on other criteria.

**Table 2:** An example illustrating alert or notification adaptation.

Task and evaluation		Completion		Notifications
**Task 1**				
	E1.1	✓^a^	Task 1 completion alert Task 2 and Task 3 are active	
	E1.2	✓	Task 1 completion alert Task 2 and Task 3 are active	
**Task 2**				
	E2.1	✓	Task 2 completion alert	
	E2.2	✓	Task 2 completion alert	
**Task 3**				
	E3.1	✓	Task 3 completion alert Task 4, and task 5 are active	
**Task 6**				
	E6.1	✓	Task 6 completion alert Module 1 completion SMS text message	

^a^✓ indicates that a user has completed the task.

#### User-Level Adaptation

As depicted in Figure 5, each task has an evaluation (*points*) associated with it. Once a user completes the task, the user obtains these points. The sum of the points obtained from each task indicates the overall score for any user. An adaptive system can have a simple adaptive rule to activate or deactivate tasks based on their overall score. For example, if we know the total score of *T_overall_* for a user, we can activate or block the availability of the next task for that user. We can use a simple rule engine, such as the following, to activate or deactivate tasks:




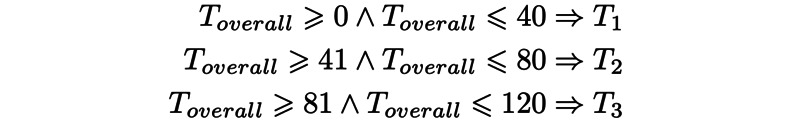




In the above rules, *T_i_* (T1, T2, and T3) is a list of tasks. In the above example, we assumed that the threshold score for each task could be decided empirically or determined by the therapists who designed the intervention. According to this example, if *T_overall_* is between 0 and 40, we would recommend Task 1 to the patient. Similarly, if *T_overall_* is between 41 and 80, we would recommend Task 2 to the patient.

### Software Quality Metrics

#### Overview

As previously mentioned, we envisioned addressing the challenge of high dropout and low user adherence in the current IDPT system. Therefore, a primary software quality metric based on International Organization for Standardization/International Electrotechnical Commission (ISO/IEC) 25000 [45] is adaptability. Moreover, based on the current IDPT system analysis, recommendations from the literature review, and discussion from domain experts, our secondary software quality attribute requirements include scalability, interoperability, security, reusability, and modifiability. We have adopted the notational convention keywords *MUST*, *MUST NOT*, *REQUIRED*, *SHALL*, *SHALL NOT*, *SHOULD*, *SHOULD* *NOT*, *RECOMMENDED*, *MAY*, and *OPTIONAL* in this section to describe these software quality attributes and compliance in the proposed RA. These keywords are to be interpreted as described in Request for Comments (RFC) 2119 [46].

#### Adaptability

We aimed to adapt interventions according to user needs and requirements to enhance user engagement and increase adherence. To adapt the intervention, we created a detailed user profile. On the basis of these profiles, we adapted the intervention. The discussion section provides several scenarios explaining how the proposed RA fulfils this need.

#### Scalability

The entire data-driven adaptive system is based on the SOA. The SOA enforces scalability by organizing services into several components that communicate over a network. Each component of the architecture can be updated and evolved in terms of hardware and software, independent of other components. For example, the IDPT intervention system server’s data storage capacity can be increased or decreased without affecting the analytics server.

#### Interoperability

Our framework supports taxonomic labeling. These are the basics of ontology. On the basis of these taxonomies, we can define several ontology codes such as *Systematized Nomenclature of Medicine–Clinical Terms, Logical Observation Identifiers Names and Codes (LOINC),* and others. The support for such taxonomies will allow us to gain interoperability. To enforce interoperability, we used *Health Level Seven International Fast Healthcare Interoperability Resources (HL7 FHIR)* as the underlying communication standard.

#### Reusability

The proposed RA uses an SOA that supports reusability to a great extent. For example, we can use the authorization server to handle authentication and authorization for several services. We can reuse the interventions for other health care treatments. Similarly, the analytics server was loosely coupled with the RA and can be reused for several different data analysis purposes.

#### Security

The authorization mechanism *must* be Transport Layer Security secured [47] and should be improved using the contemporary practices mentioned by the Internet Engineering Task Force [47]. For the prototype, the authorization is incorporated inside the IDPT system but is subject to change as a separate SOA component, similar to the authorization server used [13]. In any case, the authorization *should* issue short-lived tokens and have a mechanism open to administrators and end users to eliminate tokens in the case of a security conflict.

#### Modifiability

Modifiability incorporates *evolvability, customizability, configurability*, and *extensibility* [13]. The SOA-based architecture facilitates modifiability by allowing the manageable growth of systems [48]. These systems and components are independent of vendors, products, and technologies. This independence makes it easy to manage and modify individual components. For example, the analytics server in the architecture (described in the *Discussion* section) *may* update the ML libraries or create an additional service that consumes data and performs business intelligence without affecting other components. Similarly, the authorization server *may* create a customized interface for managing authorized clients, scopes, and permissions without broadcasting its development complexity, structure and patterns, and technological compliance with other components. However, the constituting components *must* follow a common standard for data storage and transmission.

### Expert Evaluation of the Open-Source Framework

As part of the nonempirical evaluation, we conducted an expert review. A panel of 17 experts (developers and designers) was invited to review the system and its components. We invited experts from the field, all of whom worked in the information technology industry. The review team was presented the RA and an open-source framework. An interview followed the review to determine their reaction toward the open-source framework and its components. We chose full-stack developers (7/17, 41%), front end developers (3/17, 18%), back-end developers (5/17, 29%), and system architects (2/17, 12%) with >5 years of industrial experience. The evaluation aimed to inspect the open-source framework’s modifiability, extendibility, scalability, security in authentication, reusability, and code readability. The reviewers were asked to rate the evidence of these software qualities in the presented open-source framework. The results of the expert evaluation are presented in Table 3. As shown in Table 3, experts evaluated the open-source framework as possessing most of the abovementioned capabilities. In addition to these questions, we asked open-ended questions regarding feedback, reviews, and improvements. This feedback and reviews were considered for enhancement of the open-source framework.

**Table 3:** Results of expert evaluation (N=17).

Questions	Participants, n (%)						Values, mean (SD)
	1	2	3	4	5		
Component modifiability	0 (0)	0 (0)	1 (6)	8 (47)	8 (47)	4.412 (0.599)	
Framework extendibility	0 (0)	0 (0)	4 (24)	5 (29)	8 (47)	4.235 (0.807)	
System scalability	0 (0)	0 (0)	2 (12)	4 (24)	11 (65)	4.529 (0.696)	
Security in authentication	0 (0)	0 (0)	3 (18)	9 (53)	5 (29)	4.112 (0.676)	
Component reusability	0 (0)	0 (0)	2 (12)	9 (53)	6 (35)	4.423 (0.644)	
Code readability	0 (0)	0 (0)	2 (12)	11 (65)	4 (24)	4.118 (0.582)	

### Empirical Evaluation: Case Study

In addition to nonempirical evaluation, we evaluated the framework with a small group of participants for the feasibility study for the attention-deficit/hyperactivity disorder cases in the INTROMAT project. Domain experts created a web-based intervention, and the participants were asked to interact with the intervention. The feasibility study and results are under review for publication as RCTs [49]. The feasibility study results show that the intervention system built on the top of the proposed RA can adapt to interventions, such as reminder or alert adaptation and content adaptation.

### Implication of the RA

One of the essential questions is *why the RA is essential.* Our literature review revealed a lack of standard documentation, framework, and clinical guidelines on how the IDPT system should be developed [4]. As a result, developers and researchers reinvent their own version of the IDPT system, making it more complicated, less interoperable, and lacking a common foundation. Defining RA is a well-recognized method of addressing these challenges. Martinez et al [50] mentioned that RA increases development speed, reduces operational expenses, and improves software system quality. Similarly, several other studies [6,51] have outlined the benefits of RA as it provides a template solution for a specific domain.

In the health care context, researchers, developers, and industrial partners have published RA. Therefore, one might argue *why the proposed RA is better and how it solves the identified problems.* To the best of our understanding, no RA has been reported in the psychological domain. Some related, published RAs have been compared in related work (see the *Methods* section). Furthermore, we provide a detailed architecture of the intervention system, which is part of the RA. The intervention system allows for the creation and design of interventions that can be used in several cases. Hence, both researchers and software developers can use an open-source framework or extend the framework to match their use cases. Angelov et al [52] presented a detailed framework for the analysis and design of RA. To reduce threats to validity, we used this framework [52] to analyze the proposed RA and create the contextual Table 4. We identified two problems in the current IDPT systems: (1) they lack adaptiveness, and (2) they are complex and less interoperable because of the lack of open-source standards. The *How We Propose to Increase Adaptivity in IDPT Systems* section outlines several scenarios of how the proposed RA addresses adaptiveness. We made both the RA and intervention system an open-source framework to attract researchers and developers to use it rather than reinvent it from scratch.

**Table 4:** Analysis of the proposed RA^a^ with respect to the framework presented by Angelov et al [52].

Category and questions		Details
**Context**		
	Where will it be used?	Health care providers, hospitals, and health clinics that provide digital intervention
	Who defines it?	Collaboration between psychological domain experts, software engineers, IT^b^ industry partners, and HCI^c^ experts
	When is it defined?	With a high prevalence of mental or neurological disorders in Norway and around the world, the INTROMAT^d^ project aims to provide adaptive interventions for people with mental health issues
**Goals**		
	Why is it defined?	To adapt the intervention to reduce current higher dropouts and increase user adherence
**Design**		
	What does it describe?	Components that are working together to form a data-driven adaptive system
	How is it represented?	We used a semiformal representation of RA and described each component in detail

^a^RA: reference architecture.

^b^IT: information technology.

^c^HCI: human-computer interaction.

^d^INTROMAT: Introducing Mental Health through Adaptive Technology.

### Future Work

A promising objective of this open-source framework is to adapt interventions based on user needs and preferences. However, the RA requires continuous evolution and refactoring, and so does our open-source framework for the intervention system. Our immediate future work involves (1) usability and performance evaluation of the CMS for therapists and patients’ UI, (2) evaluation of adaptive strategies and their implications on user adherence using effective RCT methods, (3) maintenance and evaluation of UIs using UI experts, and (4) building and supporting analytics server end points for adaptation. There are several potential research directions for future research with this open-source framework, including the automatic structuring of modules and tasks inside a case; taxonomic or ontology-based adaptation; interoperability; and support for better user interactions, such as adaptive conversational agents.

### Conclusions

To the best of our knowledge, this is the first study to create an RA and open-source framework for an adaptive IDPT system. The proposed RA uses a user profiling model to adapt and personalize interventions based on user needs. On the basis of the proposed RA, we created an open-source framework for an adaptive IDPT system. We followed the DDD architectural style and Test-Driven Development process to create an open-source framework prototype. We evaluated it using empirical (case study) and nonempirical approaches (SAAM method, expert evaluation, and software quality matrices). This paper presents an initial study, and preliminary evaluation results show that developers and researchers can extend the proposed RA to multiple health care interventions. Our immediate future work will involve extending and evaluating the framework for usability, performance, and other adaptive capabilities.

## Abbreviations

AAL: Ambient Assisted Living
AI: artificial intelligence
API: application programming interface
CMS: Content Management System
DDD: Domain-Driven Design
DL: deep learning
IDPT: internet-delivered psychological treatment INTROMAT: Introducing Mental Health through Adaptive Technology
ISO/IEC: International Organization for Standardization/International Electrotechnical Commission
ML: machine learning
NLP: natural language processingRA: reference architecture
RCT: randomized controlled trial
RFC: Request for Comments
SAAM: software architecture analysis methodSOA: service-oriented architecture
UI: user interface

## References

[1] (2006). Evidence-based Psychotherapy: Where Practice and Research Meet.

[2] Kelders SM, Kok RN, Ossebaard HC, Van Gemert-Pijnen JE (2012). Persuasive system design does matter: a systematic review of adherence to web-based interventions. J Med Internet Res.

[3] Mukhiya SK, Wake JD, Inal Y, Lamo Y (2020). Adaptive systems for internet-delivered psychological treatments. IEEE Access.

[4] Mukhiya SK, Wake JD, Inal Y, Pun KI, Lamo Y (2020). Adaptive elements in internet-delivered psychological treatment systems: systematic review. J Med Internet Res.

[5] Bonaccorsi A, Rossi C (2003). Why Open Source software can succeed. Res Policy.

[6] Cloutier R, Muller G, Verma D, Nilchiani R, Hole E, Bone M (2010). The concept of reference architectures. Syst Eng.

[7] Kogut B, Metiu A (2001). Open‐source software development and distributed innovation. Oxford Rev Econ Policy.

[8] (1988). Handbook of Cognitive-Behavioral Therapies, Third Edition.

[9] Kohn R, Saxena S, Levav I, Saraceno B (2004). The treatment gap in mental health care. Bull World Health Organ.

[10] (2003). Adherence to Long-term Therapies Evidence for Action.

[11] Wallin EE, Mattsson S, Olsson EM (2016). The preference for internet-based psychological interventions by individuals without past or current use of mental health treatment delivered online: a survey study with mixed-methods analysis. JMIR Ment Health.

[12] Portnoy DB, Scott-Sheldon LA, Johnson BT, Carey MP (2008). Computer-delivered interventions for health promotion and behavioral risk reduction: a meta-analysis of 75 randomized controlled trials, 1988-2007. Prev Med.

[13] Mukhiya SK, Rabbi F, Pun KI, Lamo Y (2019). An architectural design for self-reporting e-health systems. Proceedings of the 2019 IEEE/ACM 1st International Workshop on Software Engineering for Healthcare (SEH).

[14] Gary FA (2005). Stigma: barrier to mental health care among ethnic minorities. Issues Ment Health Nurs.

[15] (2020). A reference architecture for personalized and self-adaptive e-health apps. Software Architecture.

[16] (2019). On online banking authentication for all: a comparison of BankID login efficiency using smartphones versus code generators. Universal Access in Human-Computer Interaction.

[17] Connected health reference architecture. WSO2.

[18] Wartena F, Muskens J, Schmitt L, Petković M (2010). Continua: the reference architecture of a personal telehealth ecosystem. Proceedings of the 12th IEEE International Conference on e-Health Networking, Applications and Services.

[19] Rodriguez LM, Ampatzoglou A, Avgeriou P, Nakagawa EY (2015). A reference architecture for healthcare supportive home systems. Proceedings of the 2015 IEEE 28th International Symposium on Computer-Based Medical Systems.

[20] (2011). universAAL – An Open and Consolidated AAL Platform. Ambient Assisted Living.

[21] (2007). User models for adaptive hypermedia and adaptive educational systems. The Adaptive Web.

[22] (2007). Generic user modeling systems. The Adaptive Web.

[23] (2007). Adaptive information for consumers of healthcare. The Adaptive Web.

[24] (2009). Intelligent user profiling. Artificial Intelligence An International Perspective.

[25] Yogarajah A, Kenter R, Lamo Y, Kaldo V, Nordgreen T (2020). Internet-delivered mental health treatment systems in Scandinavia - a usability evaluation. Internet Interv.

[26] Inal Y, Wake JD, Guribye F, Nordgreen T (2020). Usability evaluations of mobile mental health technologies: systematic review. J Med Internet Res.

[27] (2003). Domain-Driven Design Tackling Complexity in the Heart of Software.

[28] (2000). Test-driven Development By Example.

[29] Mukhiya SK, Rabbi F, I Pun VK, Rutle A, Lamo Y (2019). A GraphQL approach to Healthcare Information Exchange with HL7 FHIR. Procedia Comput Sci.

[30] - (2020). Sakimura, J. Bradley, M. B. Jones, B. Medeiros, and C. Mortimore, “Final: OpenID Connect Core 1.0 incorporating,”.

[31] Mukhiya SK, Ahmed U, Rabbi F, Pun KI, Lamo Y (2020). Adaptation of IDPT system based on patient-authored text data using NLP. Proceedings of the 2020 IEEE 33rd International Symposium on Computer-Based Medical Systems (CBMS).

[32] Sharma A, Choudhury M, Althoff T, Sharma A (2020). Engagement patterns of peer-to-peer interactions on mental health platforms. Proceedings of the International AAAI Conference on Web and Social Media.

[33] Bremer V, Chow PI, Funk B, Thorndike FP, Ritterband LM (2020). Developing a process for the analysis of user journeys and the prediction of dropout in digital health interventions: machine learning approach. J Med Internet Res.

[34] Nemesure MD, Heinz MV, Huang R, Jacobson NC (2021). Predictive modeling of depression and anxiety using electronic health records and a novel machine learning approach with artificial intelligence. Sci Rep.

[35] (2016). Microservice Architecture Aligning Principles, Practices, and Culture.

[36] Zukerman I, Albrecht DW (2001). Predictive statistical models for user modeling. User Modeling User Adapt Interact.

[37] Lieberman H, Fry C, Weitzman L (2001). Exploring the Web with reconnaissance agents. Commun ACM.

[38] Höök K, Karlgren J, Wærn A, Dahlbäck N, Gustaf Jansson C, Karlgren K, Lemaire B (1996). A glass box approach to adaptive hypermedia. User Model User Adap Inter.

[39] An D, Carr M (2017). Learning styles theory fails to explain learning and achievement: recommendations for alternative approaches. Personality Individual Diff.

[40] (2020). Using responsive web design to enhance the user experience of chronic disease management portals for clinical users. Delivering Superior Health and Wellness Management with IoT and Analytics.

[41] Hofmeister C, Kruchten P, Nord RL, Obbink H, Ran A, America P (2007). A general model of software architecture design derived from five industrial approaches. J Sys Softw.

[42] Kazman R, Bass L, Abowd G, Webb M (1994). SAAM: a method for analyzing the properties of software architectures. Proceedings of 16th International Conference on Software Engineering.

[43] Kazman R, Klein M, Barbacci M, Longstaff T, Lipson H, Carriere J (1998). The architecture tradeoff analysis method. Proceedings of the Fourth IEEE International Conference on Engineering of Complex Computer Systems (Cat. No.98EX193).

[44] Kobsa A, Koenemann J, Pohl W (2001). Personalised hypermedia presentation techniques for improving online customer relationships. Knowl Eng Rev.

[45] - (2012). -. -.

[46] Key words for use in RFCs to indicate requirement levels. RFC Editor.

[47] (2015). Recommendations for secure use of Transport Layer Security (TLS) and Datagram Transport Layer Security (DTLS). RFC Editor.

[48] Reference model for service oriented architecture 1.0. OASIS Standard.

[49] Nordby ES, Kenter RM, Lundervold AJ, Nordgreen T (2021). A self-guided internet-delivered intervention for adults with ADHD: a feasibility study. Internet Interv.

[50] (2013). Benefits and drawbacks of reference architectures. Software Architecture.

[51] Towards a domain specific software architecture for scientific data distribution. SIP Commons.

[52] Angelov S, Grefen P, Greefhorst D (2012). A framework for analysis and design of software reference architectures. Info Softw Technol.

[53] Intromat homepage. Intromat.

